# Photonic sintering of copper for rapid processing of thick film conducting circuits on FTO coated glass

**DOI:** 10.1038/s41598-023-32044-2

**Published:** 2023-03-28

**Authors:** Bahaa Abbas, Eifion Jewell, Yin Cheung Lau, Justin Searle, Tim Claypole

**Affiliations:** Faculty of Science and Engineering, Swansea University, Swansea, UK

**Keywords:** Materials science, Nanoscale materials, Electronic properties and materials

## Abstract

Copper potentially provides a cost-effective replacement for silver in printed electronic circuitry with diverse applications in healthcare, solar energy, IOT devices and automotive applications. The primary challenge facing copper is that it readily oxidizes to its non-conductive state during the sintering process. Photonic sintering offers a means of overcoming the oxidation by which rapid conversion from discrete nano-micro particles to fully or partially sintered products occurs. An experimental study of flash lamp sintering of mixed nano copper and mixed nano/ micro copper thick film screen printed structures on FTO coated glass was carried out. It shows that there may be multiple energy windows which can successfully sinter the thick film copper print preventing detrimental copper oxidation. Under optimum conditions, the conductivities achieved in under 1 s was (3.11–4.3 × 10^–7^ Ω m) matched those achieved in 90 min at 250 °C under reducing gas conditions, offering a significant improvement in productivity and reduced energy demand. Also present a good film stability of a 14% increase in line resistance of 100 N material, around 10% for the 50N50M ink and only around 2% for the 20N80M.

## Introduction

Copper potentially offers an alternative to silver in printed circuity which is the basic fundamental part of any electrical/electronic device^1^. While silver has excellent conductivity, is stable in organic suspensions and has good lifetime it is not only more expensive but also subject to fluctuations in price. There is increasing interest in the use of copper as a potential printed conductive material with several options being explored including copper precursors, copper salts, copper oxide particles, nano copper and micro copper formulations^1,2^. Of these, copper particulate inks provide many favourable characteristics with similar rheology and printing behaviour to their silver particle counterparts. One challenge is to form particles that do not quickly transform into electrically insulating copper oxide during processing, which reduces their electrical conductivity^3^. The most common means to prevent oxidation is to thermally sinter (150–400 °C) the materials in an inert, vacuum or ideally reducing atmosphere,^4,^^5,6^. However, traditional thermal oven processes are generally inherently a batch process with 60–120 min process times. This limits production rates and also possess an inherently high carbon footprint directly associated with the elevated temperature, long processing times and indirectly associated with the production and storage of the reducing atmosphere gas. Wide area flash lamp photonic sintering has been widely used for sintering silver nano inks^7,8^^–^^10^, nickel nano inks^11^ as well as processing other complex nano materials and devices^12,13^. In addition to the fast sintering, the main advantages of the photonic sintering technology, it potentially sinters without causing any damage to the substrate and eliminates the need for a reducing atmosphere to be employed^14^. This significantly reduces processing times and reduces the overall energy demand of the sintering process.

There have been multiple reports of photonic sintering being used on copper conductive inks. Photonic sintering has been widely shown to be able to sinter thin inkjet printed copper nano particle inks on polymer substrates resulting in conductivities equivalent thermal sintering under protective atmospheres^9,15,16^. Literature on the thicker films (> 5 µm) which are typically produced by screen printing is sparse. The thick film nature of the substrate provides an additional quantity of material which needs to be sintered and a provides the additional challenge of transferring the energy through the bulk to the core of the film from the exposed surface. A comparison of thermal and laser sintering of screen printed copper nanoparticles ink under a formic acid reducing atmosphere on PI substrate showed that the laser sintering process under a controlled laser power and scan speed could achieve almost the same resistivity (1.41 × 10^−5^ Ω cm) as that achieved with thermal sintering (1.30 × 10^−5^ Ω cm) under the nitrogen atmosphere^6^. This highlights the feasibility photonic sintering of thicker films, although the study was not carried out with a flash lamp and was undertaken on a polymer substrate. Being a broad area single exposure process, flash lamp sintering has an advantage over laser sintering of potentially being able to instantly process a larger area.

Several important applications involve the use of coated glass as a substrate such as Perovskite PV^17,18^ and smart windows^19,20^ where digital control of transmission is required for heat management or user privacy. This provides additional complications and challenges for photonic sintering. A portion of the photonic energy may be absorbed by the coating, the photonic energy may be absorbed of the glass and there is an appreciable energy sink^21,22^ associated with the mass of the glass (3–10 mm compared to 100–250 µm of polymer). In addition, the thermal shock experienced by each material (the coating and glass are brittle) and their relative thermal expansion may impact film integrity and inter-layer adhesion. FTO (Fluoride doped Tin Oxide) is a popular coating applied to the glass offering conductivity and transparency at a lower cost compared to ITO (Indium doped Tin Oxide). Depending on the relative lamp emission and FTO absorption spectra, which can vary with film structure and texture^23^, the presence of the FTO can alter the heating distribution and rate within the copper printed structure. For example, enhanced absorption could potentially act as an additional energy source at the boundaries of any feature possibly highlighting issues related to printed feature geometry.

There literature has highlighted that photonic sintering has potential for copper sintering, but there is lack of knowledge pertaining to glass substrates that have been coated with an FTO surface or a thick screen-printed film. Thus, the aim of the study was to examine the feasibility of photonically sintering a thick film of copper ink on a FTO glass substrates and whether it offers potential improvements over conventional reducing atmosphere oven sintering.

## Method

**Figure 1:**
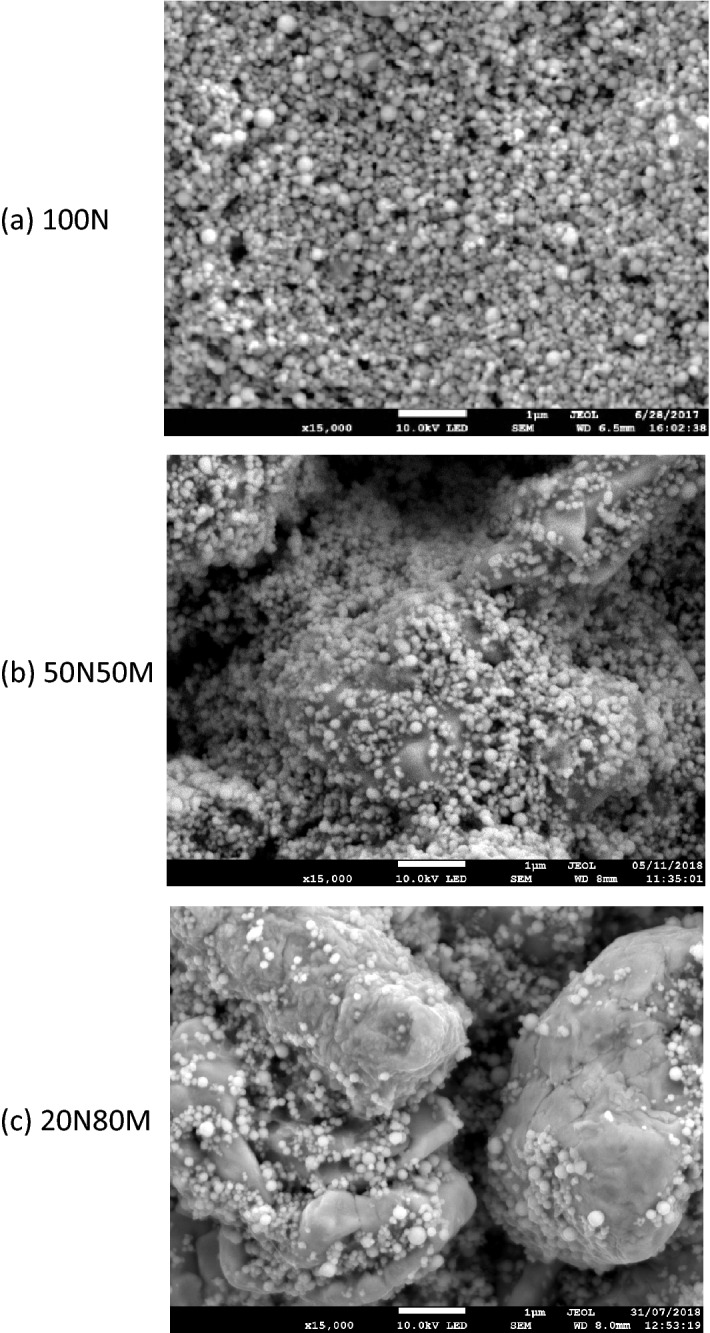
Dry screen-printed inks.

Three copper materials were sourced from Intrinsiq materials, these were labelled 100N, 50N50M & 20N80M in accordance with the proportion of nano (N) and micro (M) particle, Table 1 & Fig. 1. The copper materials were custom manufactured variations of commercial materials (CP-001) utilising a controlled quantity of nano and micro copper. The materials were produced by Intrinsiq Materials Ltd using their patent copper particle production processes which can produce nano and micro materials. These were blended and mixed with the proprietary binder and solvent. No attempt was made to investigate the material using laboratory analytical techniques, although further rheological and TGA data has been published^4^.

**Table 1:** Copper ink characteristics.

	100N	50N50M	20N80M
Copper loading (% by mass)	76.2	74.6	79.2
Particulate	Nano	Nano: Micro	Nano: Micro
Micro : nano particle %	Nano only	50:50	20:80
Primary solvent BP (°C)	117	136	136

**Figure 2:**
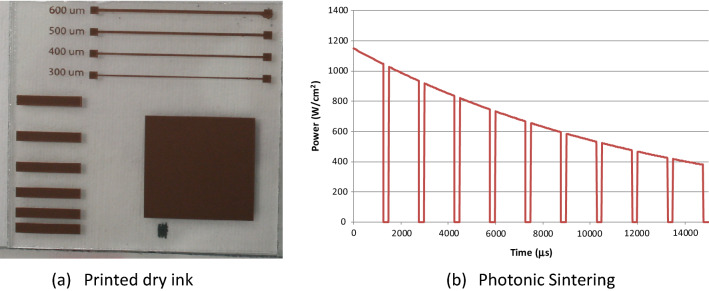
Printed dry but un-sintered image (**a**) and (**b**) and an example of a 10-pulse photonic energy profile (energy density of 8.9 J/cm^2^).

Each ink was screen printed using an image, Fig. 2, which consisted of areas which allowed for characterization of fine lines (200–600 µm wide). The images were printed to Tec-7 FTO coated glass from NSG (Nominal sheet resistance of 7 Ω/sq), with a 110–34 polyester mesh on an automatic ATMA flat bed printing machine. In addition, the 100 N material was printed through a 61/64 polyester mesh in order to vary film thickness.

An initial investigation study was carried out to see if the photonic curing could be used to both dry and sinter the wet ink. But this was rapidly discarded as a concept as the photonic process had minimal effect on the wet ink, even at its highest setting. The energy required to volatilize the solvent and the kinetic limitations of migration of the solvent through the wet ink to film surface were seen as mechanisms for this behaviour. Subsequently, all samples were dried thermally in a Thieme hot dryer at 80 °C for a residence time of 15 min in accordance with manufacturer’s recommendations. This allowed solvent evaporation to occur such that a dry film was obtained without the excessive temperature where the nano-particle encapsulant would undergo sublimation. Photonic sintering was carried out using a PulseForge 1200 photonic curing system which creates the high light intensity by controlled discharging of a large capacitor bank through the flash lamp. The characteristic energy output follows a decay as the stored charge is discharged through the lamp, superimposed with pulsed nature of the lamp output, Fig. 2.

The total energy output of each pulse was determined by the voltage, pulse duration and the duration time employed. In practice, the initial charge state, length of each pulse, the number of pulses and pulse interval can be varied independently (within limits) to result in the same total energy input but from distinct pulse irradiation profiles. However, the total energy input provides a meaningful, if somewhat simplistic, metric for analysis which has been used in literature. In order to examine the extremes of the operational window, a 3 pulse and 10 pulse strategy was adopted from preliminary testing.

Each sample was placed in the middle of the sintering bed (approximately 250 × 250 mm) such that illumination was constant each time. The charging of the capacitor and the cooling of the bed meant that samples were sintered at around three minutes intervals. The resistance of the pattern structures was characterised with a Megger MIT 330-EN digital low resistance multi-meter. As the copper is printed to the FTO, the measurement of line resistance therefore considers the parallel resistance of the printed ink and the underlying conductive FTO. Contact resistance and sheet resistance were calculated using the TLM method^24^. A Rofin 1064 nm Nd-YAG laser scribe was used for removal of the FTO to isolate the TLM area of the print. Three samples were produced at each condition and a mean taken. Within the three samples, line resistances were ± 0.02 Ω, sheet resistance 0.04 Ω /sq and contact resistance were ± 0.44 Ω. The film thickness was measured using a Talysurf surftronic 100 stylus profileometer without signal filtering. Absolute resistivity (ρ) and relative resistivity (ρ/ρ_Cu_) was calculated from the measured film cross sectional area (A) and the resistance (R) over the length (l) using R = rl/A. The optimized sintering for each material under controlled atmosphere thermal sintering conditions^4^, were used as reference to examine relative performance of the photonic sintering.

## Results

**Figure 3:**
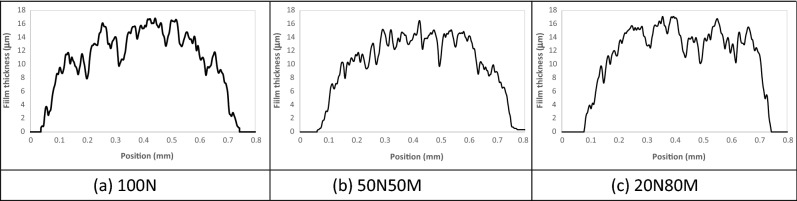
Cross sectional topologies of the 600 mm printed line.

The inherent difficulty associated with photonic curing is that optimization of the photonic regime is required. Excessive peak intensities lead to delamination of the ink from the substrate, while insufficient power does not result in any sintering of the copper. Through a process of refined pulse width, power and spacing the nominal optimum conditions were established for the ink/substrate combination. This trial and error approach was necessary, but also wasteful in the number of samples required. At each condition, the test was “destructive” in that even if the sample showed no visible sign of sintering, it would not be used as a test sample again. Typically, around 80–100 samples of each material were required to focus in on a set condition which could be considered optimized. During this optimization process the 600 µm line was used as a measurement reference, with visual observations of the other printed features in order to refine exposure conditions. The film profiles for the nominally 600 mm line show that the topology of the printed lines is similar between materials, Fig. 3. Peak thickness is around 16 µm with mean thickness of around 12 µm and each nominally 600 µm line prints at mean width of 720 µm ± 20 µm. Evident in the cross section of the lines is some mesh marking which arises from withdrawal of the screen during print process and is typical of the pattern seen when printing highly viscous materials^25^.

For each number of pulses, there exists an optimum sintering condition (lowest resistance) that can be achieved. The inference from this is that there is a minimum pulse energy related to each material to achieve a lower resistance. The energy available for the generation of light is dependent on the size of the capacitors and the voltage applied to that capacitor^26–28^. An initial optimization study was carried out to identify an operational range of photonic energy profile. A strategy was employed to obtain the best possible effect of the flashlight which could lead to a good conductivity range by applying a different number of pulses with variety of voltage which then enabled the estimation of the maximum, and the minimum power applied. Through, the refined pulse width, power and spacing the nominal optimum conditions were established for the ink/substrate combination. Starting from the same initial capacitor charge, a single pulse did not provide enough energy input to sinter and as such a 3–10 pulse strategy was adopted after several trials carried out and according to the machine safety operation and energy capacity supplied by the manufacturer.

**Figure 4:**
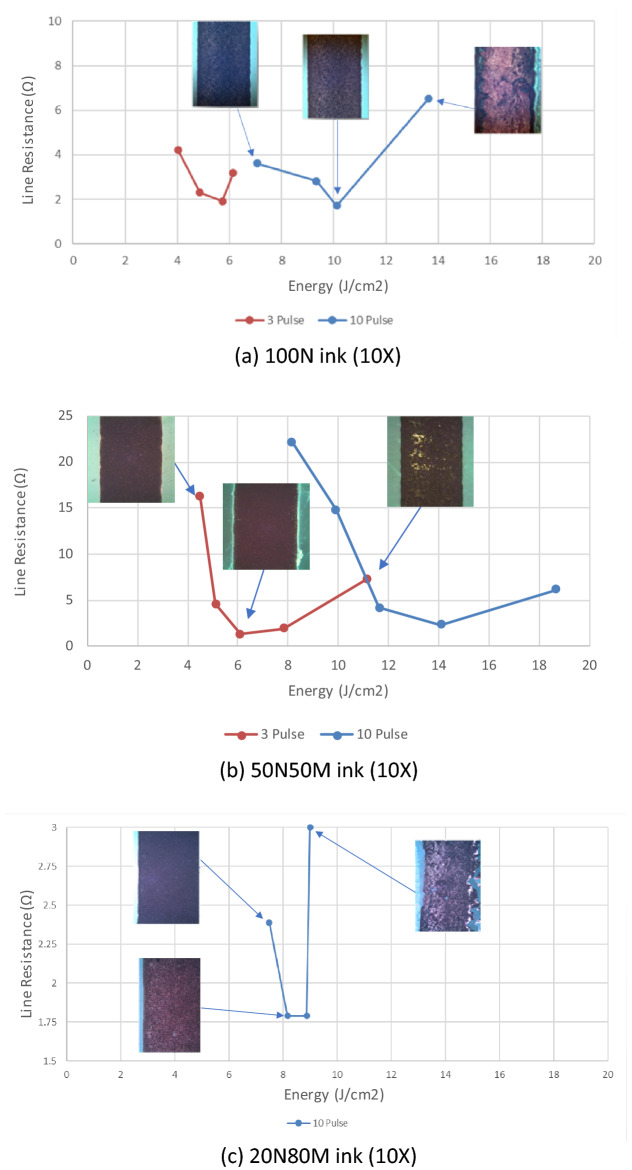
Sintering envelopes for the three inks defined by the resistance of the 600 µm wide line.

Figure 4 shows several examples of the relationship between the total energy input (integrated from each pulse duration) and the net line resistance measured for the 600 µm wide line. For the 100N and 50N5M materials, near identical resistances can be obtained at lower energies, but the 10 pulse provided a wider operational window. The energy per pulse decay observed in Fig. 2, suggests that the impact of a larger number of pulses diminishes but may have a contributory impact on reducing adverse thermal stresses on the film.

Examination of the data set provided by the experimental study shows that it is possible to achieve electrical performance from the optimised photonically cured material which is comparable with the results obtained through thermal oven sintering for the micro particle containing materials, Table 2. The pure nano particle material exhibits similar absolute performance but the performance relative to the oven sintered nano material is lower by between a factor of 3 and 5. Thus, while materials containing micro particles can be readily photonically sintered (from a purely conductive perspective), photonic sintering of pure nano materials results in a penalty compared to thermal sintering.

**Table 2:** Absolute resistivity relationship for each material for the 600 µm line.

Ink	ρ (abs Resistivity) × 10^–7^ Ω m	Relative to bulk copper	Relative to best thermal^4^
100N	3.35	10–12	3–5
50N50M	3.11	12	1
20N80M	4.3	13	1

**Figure 5:**
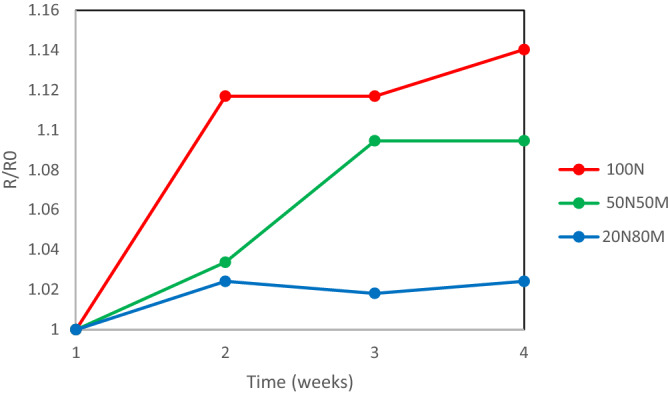
600 µm Line resistance change over time in weeks for each photonically sintered ink at the optimum sintering condition.

Stability measurements over a period of 4 weeks for the optimised samples shows a 14% increase in line resistance of 100N material, around 10% for the 50N50M ink and only around 2% for the 20N80M material which contains the minimum quantity of nano-particles, Figure 5. This suggests that there are either nano-particles within the film which have not fully sintered and remain active or the open porosity of the film allows for greater exposure of the air for oxidation to occur.

**Figure 6:**
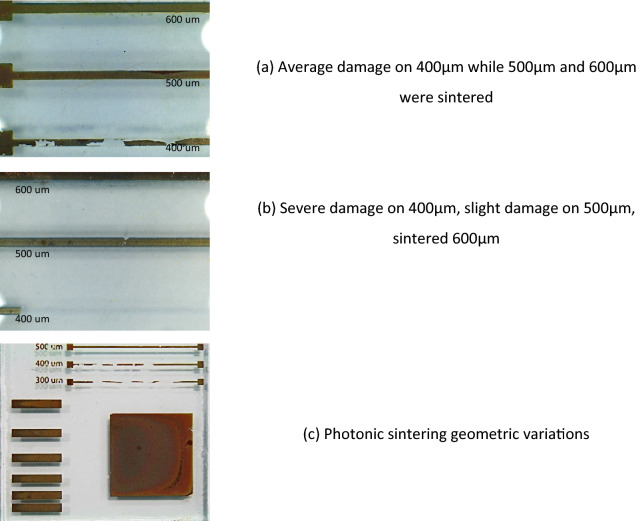
The impact on feature size on the sintering conditions for the 100 N ink.

As surface absorption was the source of energy for the sintering process, then some changes in the optimum sintering conditions were expected for each line width as the ratio of surface area to bulk volume changes. This was experienced with finer structures being adversely affected by exposure to the photonic energy with areas exhibiting substrate delamination and physical damage, Fig. 6a,b. The reliance of the optimum sintering conditions on the geometric nature of the feature being sintered is also evident in Fig. 6c where the finer features have been ablated, although the central square displays regions which remain un-sintered.

**Figure 7:**
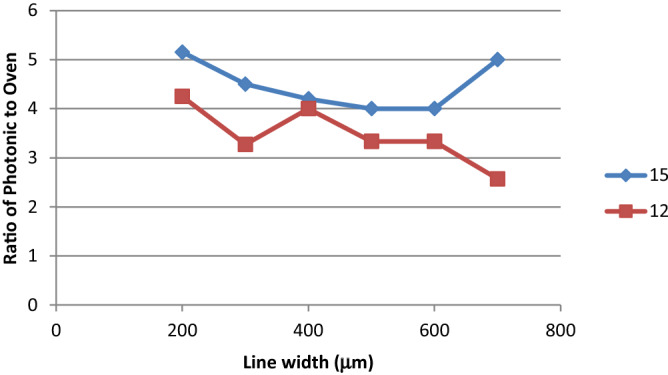
The impact of feature sizes and film thickness on the relative conductivity between the oven sintered and the photonically sintered printed lines for the 100N material.

Thus, the ideal photonic sintering conditions are related not only to the material properties and film thickness, but also to the feature size being printed. This is evident in Fig. 7 where the relative conductivity ratio between thermal oven sintered samples and the photonically cured samples is illustrated for the 100N material. Under a given set of photonic sintering conditions optimised for the 600 mm lines for the 100N material, thinner lines see an increase in resistance compared to their oven sintered counterparts. Thinner features benefit from a larger surface for photonic absorption per unit volume compared to the thicker lines, but the difference is minimal (around 8% over the 200–700 µm range). However, these line edges are often the source of defects associated with over exposure during the sintering process. This is true for films printed at a mean thickness of 12 µm (110–34 mesh) and a 15 µm film (61/64 mesh). The thicker film provides lines whose resistance is higher compared to their oven sintered counterparts. This result is in line with expectations in that the thicker film is more challenging to sinter to the same degree.

The improved performance of the photonic sintering with the thinner film is likely related to two primary mechanisms the depth of energy penetration and the thermal mass. With the thinner film, the initial transformation to a highly reflective copper surface then becomes an optical barrier to the absorption of the energy of subsequent layers. The process thus becomes self-limiting in terms of its ability to transfer energy to the material at substrate interface with a detrimental effect on the upper surface of the film or delamination of the film. With the thicker film, the lower portions of the film act as a thermal sink taking that energy which is absorbed in the upper surface. If the energy taken does not raise the nano-copper beyond its sintering temperature, then there is no subsequent change in structure.

These finding has significant implications for circuit designer as it limits the variety of dimensions which can be used within a circuit, if circuits are to be photonically sintered. Multiple feature sizes will likely result significant variation in the levels of sintering depending upon the feature size, with larger features having a tendency to under sinter when compared to smaller features.

**Figure 8:**
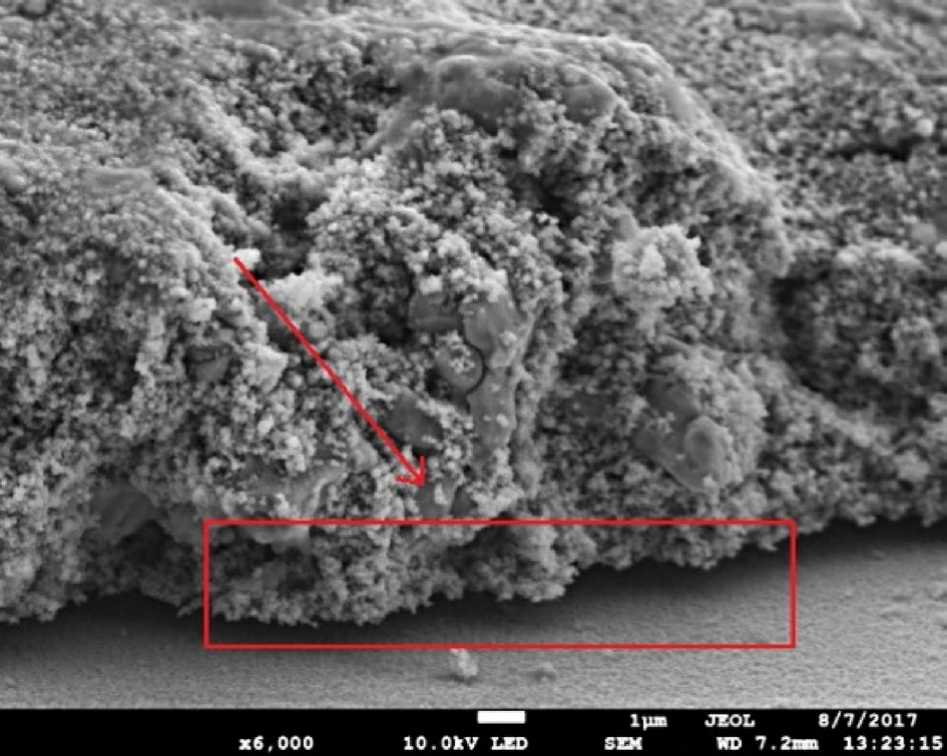
FTO—100 N Interface when photonically sintered.

The adhesion performance of the pure nano-particles material is significantly poorer than micro-particle containing materials (Table 3). The 100 N ink possessed a 0B rating in terms of scratch resistance and the application of tape removed all the material from surface, similar adhesion results observed with thermal sintering^4^. A possible explanation is that the multi-pulse sintering causes a volumetric reduction to the particle size^29,30^. There may be rapid evaporation of the binder and the inter nano-particle sintering which induces a compressive stress in the film which overcomes the ink/substrate adhesion. Also, the rapid sintering process causes a thermal shock on the interface and due to the relative coefficient of thermal expansion (9 × 10^–6^/°C for glass compared to 17 × 10^–6^/°C for bulk copper) stress is induced at the interface, Fig. 8. Increasing the micro particle component improved the scratch resistance with a 5B scratch resistance being achieved for the 20N80M ink and removed very little material from the FTO glass with a tape.

**Table 3:** Adhesion tape test result.

Ink (nano-micro)	Sintering energy (J/cm^2^)	0–5B	Tape after test
100N	10.1	0B	
50N50M	6.1	1–3B	
20N80M	8.9	5B

**Figure 9:**
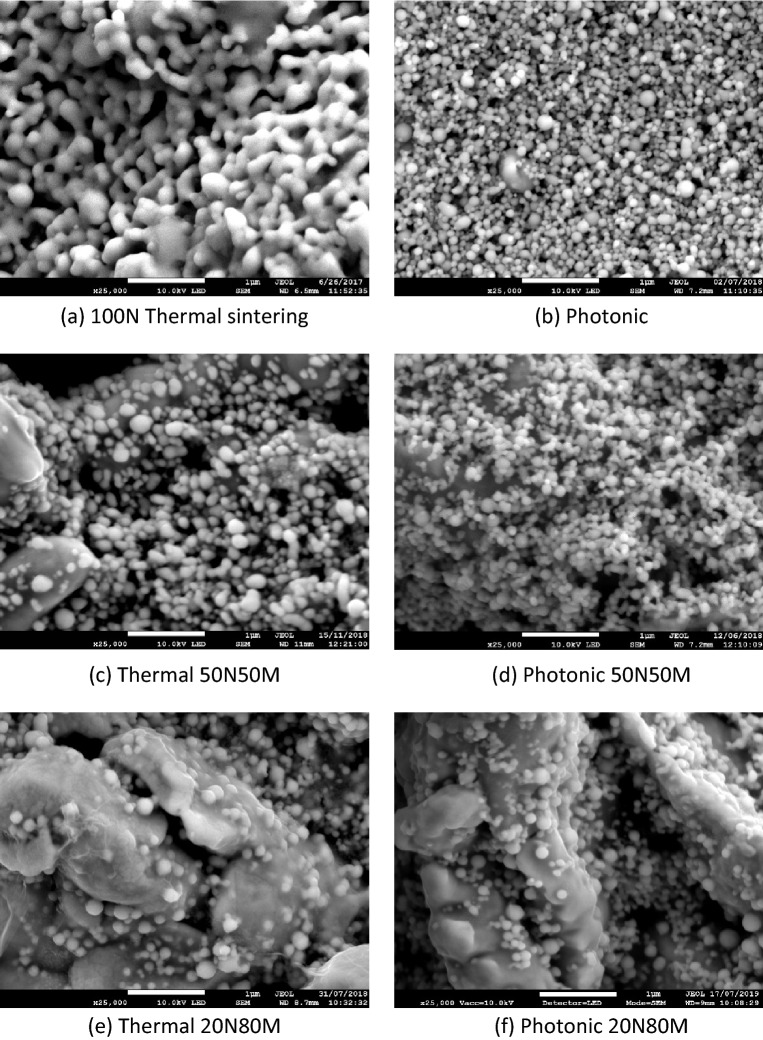
SEM images at × 25,000 for each material at its optimum thermal and photonic sintering condition. Thermal sintering in (**a**), (**c**) and (**e**) corresponds to 200 °C, 90 min in Formic acid reducing atmosphere. Photonic (**b**), (**d**) and (**f**) correspond to the optimum conditions (lowest resistance) obtained photonically.

The difference in the photonic sintering can be explained by comparing the microstructure of the materials, Fig. 9. Thermal sintering of the 100N material results in a structure where each particle merges with its near neighbours, Fig. 9a, providing a ready route for charge transfer through the structure^4^. The porosity of the film results in lower conductivity than that of pure copper, but the impact of particle–particle resistance is minimized. There is little evidence of such intimate merging between the particles when photonically sintered with each discrete nano-particle touching, but not merging at their edges. There is a less significant difference between the microstructure of the photonic and thermally sintered particles when the micro particles are present, Fig. 9c,d,e,f. In each instance the nano-particles sinter to the micro particles. The conductive path through the micro/nano blends remains predominantly through the micro particles with some additional contact being supplied by the nano particles.

**Figure 10:**
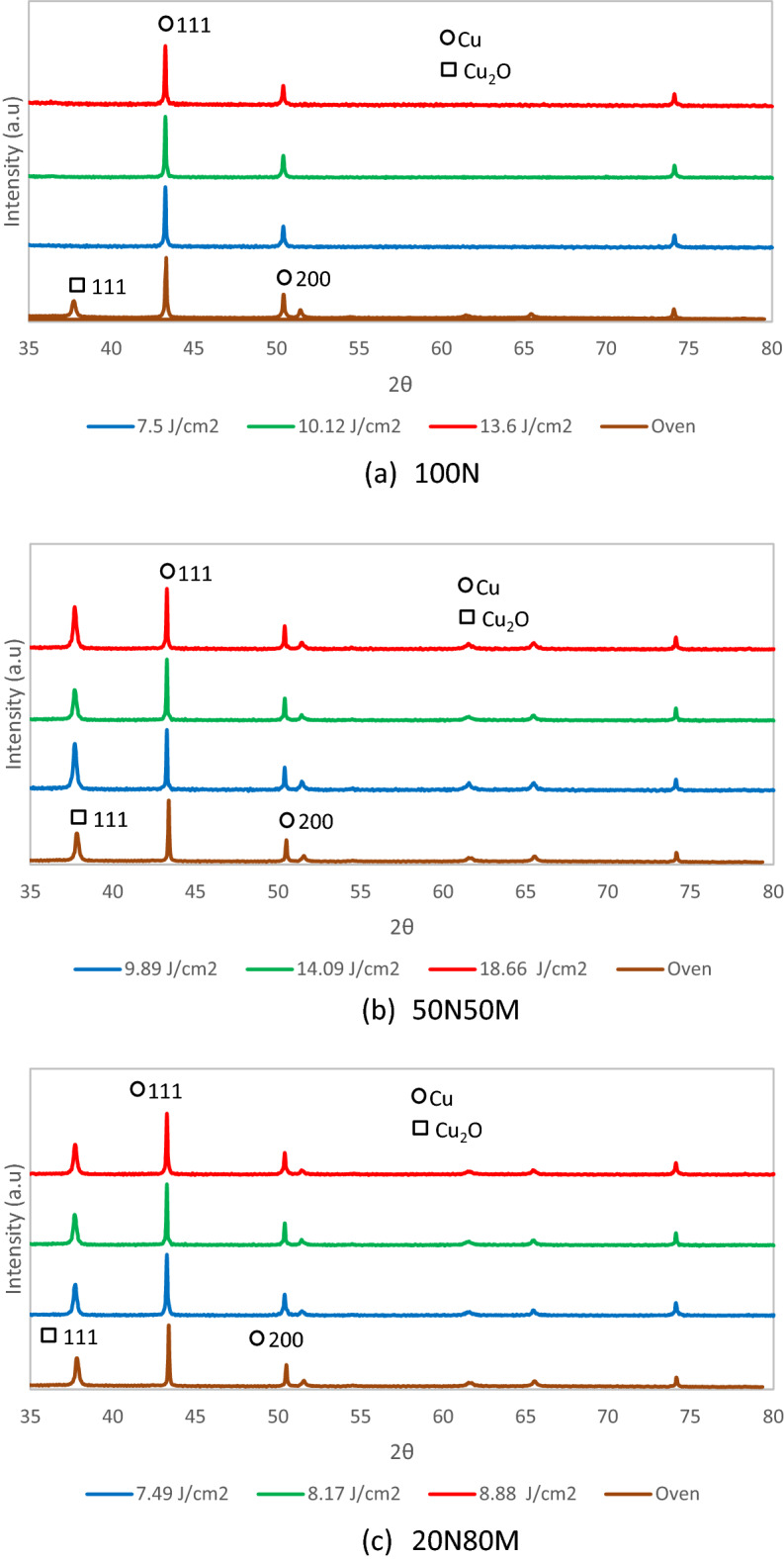
XRD of the (**a**) 100 N, (**b**) 50N50M & (**c**) 20N80M for the 600 µm wide line.

X-ray diffraction of the 100N ink shows that diffraction peaks with strong intensities appear at angles corresponding 2Ɵ 111, 200 and 220, indicating the formation of metallic copper with minimal oxidation all energy settings applied^12^ , Fig. 10a. At first glance, this can be considered beneficial in comparison with oven sintering where some oxidation is seen. However, the lower conductivity of the photonically cured material does not reflect this. It is postulated that the capping shell, in which the copper nanoparticles are initially encased, is not completely ejected from the structure during the short time scale exposure by the lamp. Subsequently, the nano particles are unable to neck at the junction between particles and create greater contact area for charge transfer, Fig. 9a,b. This highlights the difficulty obtaining a pulse energy / form where the capping agent is just removed and the particles neck without the energy absorption having a destructive impact on integrity of the film. This would also explain the increase in resistance seen with sample ageing where a slower oxidation process occurs as oxygen slowly diffuses through capping agent. The 50N50M material shows similar characteristics, which appear to be independent of the sintering process, Fig. 10b, with similar levels of oxide formation. This behaviour is echoed by the 20N80M material where copper oxide is again formed, almost independently of the sintering method, Fig. 10c.

## Discussion

Materials containing micro particles exhibit almost identical electrical performance characteristics and essentially have a similar chemical structure and physical robustness (adhesion), whatever the sintering method used. Photonic sintering of the microparticles therefore provides a viable manufacturing methodology offering 3 orders of magnitude reduction in processing times (< 10 s) compared to a minimum of an hour (including cool down period) by thermal means. The additional requirement reducing gasses and lower temperatures makes an additional case for photonic processing offering lower operational costs as well improved safety. There may also be some significant improvement in lower manufacturing carbon footprint, but this needs consideration of whole system efficiency as well as lamp efficiency. The absolute performance of the microparticle containing inks is below that observed with pure nano particle inks, but the wider operational window and stability of the materials makes them attractive for process consistency. A material made from pure nano copper has conductivity benefits when thermally sintered, but performs poorly in terms of physical characteristics when photonically sintered.

There remains considerable work to be down on the scalability of the lamp system and the structural integrity of the glass when subjected to photonic illumination at a larger scale. Localised stresses induced by the differential absorption of the FTO coated glass and the copper ink does not appear to be a problem, but larger macro temperature differences induced by the photonic sintering process would need to be examined from a practical perspective.

## Conclusions

It is possible to photonically sinter the screen-printed thick copper films on FTO glass substrates. There is a significant interaction between the material properties at a nano and micro scale and the photonic energy required for successful sintering of the screen-printed copper. There is a reduction in conductivity by a factor of 5 and 7 times for the pure nano-particle ink when photonically sintered and this can be associated with the lack of nano-particle sintering within the film. The operational window for sintering is small for the purely nano material with lamp output energy levels slightly below the window failing to sinter the film, while catastrophic failure occurs above the upper limit. The operational window of the nano/micro particle blends is wider, and the resultant conductivity is close to the pure nano material, but offering improved adhesion performance. This offers a means of sintering without controlled gas environments within seconds, when compared to hours in thermal sintering. The ideal photonic exposure conditions are linked to the feature size and film thickness, and this imposes a critical interaction between the processing and design stages. Further work is recommended on establishing design rules which relate print and material characteristics to the emission profile required. However, the optimum sintering conditions vary with the feature size which limits the flexibility of the circuit design.

## Data Availability

The datasets generated and analysed during the current study are available from the corresponding author on reasonable request.
